# Virtual reality to understand pain-associated approach behaviour: a proof-of-concept study

**DOI:** 10.1038/s41598-023-40789-z

**Published:** 2023-08-23

**Authors:** Kirsten Hilger, Anne-Sophie Häge, Christina Zedler, Michael Jost, Paul Pauli

**Affiliations:** Department of Psychology I, University of Würzburg, Marcusstr. 9-11, 97070 Würzburg, Germany

**Keywords:** Human behaviour, Anxiety

## Abstract

Pain-associated approach and avoidance behaviours are critically involved in the development and maintenance of chronic pain. Empirical research suggests a key role of operant learning mechanisms, and first experimental paradigms were developed for their investigation within a controlled laboratory setting. We introduce a new Virtual Reality paradigm to the study of pain-related behaviour and investigate pain experiences on multiple dimensions. The paradigm evaluates the effects of three-tiered heat-pain stimuli applied contingent versus non-contingent with three types of arm movements in naturalistic virtual sceneries. Behaviour, self-reported pain-related fear, pain expectancy and electrodermal activity were assessed in 42 healthy participants during an acquisition phase (contingent movement-pain association) and a modification phase (no contingent movement-pain association). Pain-associated approach behaviour, as measured by arm movements followed by a severe heat stimulus, quickly decreased in-line with the arm movement-pain contingency. Slower effects were observed in fear of movement-related pain and pain expectancy ratings. During the subsequent modification phase, the removal of the pain contingencies modified all three indices. In both phases, skin conductance responses resemble the pattern observed for approach behaviour, while skin conductance levels equal the pattern observed for the self-ratings. Our findings highlight a fast reduction in approach behaviour in the face of acute pain and inform about accompanying psychological and physiological processes. We discuss strength and limitations of our paradigm for future investigations with the ultimate goal of gaining a comprehensive understanding of the mechanisms involved in chronic pain development, maintenance, and its therapy.

## Introduction

Chronic pain is a prevalent, complicated, and serious problem. It has a severe impact on the sufferers’ everyday life, psychological well-being, and a noticeable influence on society as a whole^1,2^. In chronic pain conditions, the pain has lost its protective function and becomes a burden by itself; often resulting in disability. In the context of musculoskeletal pain, the Fear-Avoidance Model suggests the emergence of a vicious circle of fear, approach-avoidance conflicts, and pain after an acute or potential tissue damage. This vicious cycle is hypothesized to be maintained due to a self-reinforcing mechanism^3–5^.

Fear of movement-related pain, pain expectancy, and overt approach-avoidance behaviour can be examined in controlled laboratory settings. For instance, avoidance behaviour was investigated by introducing the possibility to escaping a painful stimulus and by (negatively) reinforcing this behaviour^6^. In addition, extinction, generalization, reinforcement, and goal conflicts were demonstrated in experimental investigations^7–11^. Classical conditioning^12^ was suggested to play a critical role in the acquisition, generalization, and extinction of fear of movement-related pain^13^, while operant learning of avoidance behaviour was proposed to be the crucial factor for the persistence of chronic pain and resulting disability^14^. From a classical conditioning perspective, pain can be considered as an unconditioned stimulus (US) and the (proprioceptive and visual feedback about the) movement as a conditioned stimulus (CS). The initially neutral CS gains a fear association after multiple pairings with the US. In contrast, operant or instrumental learning characterizes learning about the consequences of one’s own behaviour^15^. Such operant learning mechanisms might explain the persistence of chronic pain as successful avoidance of movements is negatively reinforced by the absence of pain.

Experimental paradigms have been developed to investigate respective associative^16^ and operant^10^ mechanisms within controlled laboratory settings. To ensure sufficient ecological validity of experimental paradigms, three aspects require specific consideration: First, in reality, avoidance is mostly accompanied by non-neglectable costs (e.g., loss of social contacts in chronic pain^14^). Thus, introducing realistic costs for reduced approach evolved as a critical factor in laboratory research (e.g., muscle effort^10^). Second, in chronic pain, pain-related avoidance behaviour is learned without any explicit instructions. This is not always easy to realize in laboratory research but might also represent a crucial factor to be considered in the development of experimental paradigms (for a good example, see Ref.^10^). Finally, anticipatory fear reactions to movement-related pain become visible across multiple dimensions (e.g., psychological, physiological) that can be assessed using different methodologies (e.g., verbal fear and expectancy ratings, avoidance behaviour, electrodermal activity; see Ref.^17^). To gain a holistic understanding of the mechanisms underlying pain-related behaviour, experimental paradigms should therefore allow for simultaneous assessment of behavioural, psychological, and physiological components.

In recent years, Virtual Reality (VR) developed as a promising tool for pain research and its treatment^18–21^. VR creates a simulated 3D environment typically displayed using head-mounted displays (HDM) or wallscreen projections. These techniques aim to achieve presence, i.e., the feeling of being physically present in the virtual world^22,23^. Recent pain research demonstrated that VR can not only modulate pain but also affect pain-related physiological factors. For instance, VR has been found to induce clinically significant pain reductions in chronic neck pain (e.g., Ref.^24^) and to alleviate movement-related fear in veterans with chronic pain^25^. In the context of acute pain, the analgesic effects of immersive VR have been related to smaller increases in brain activity in regions associated with pain stimuli and higher pain tolerances through the modulation of autonomic responses^26,27^.

This proof-of-concept study introduces a new Virtual Reality paradigm to the study of pain-associated approach behaviour. Specifically, the paradigm combines three-tiered heat pain stimulation with three types of arm movements. It does not use any explicit instructions to approach or avoid, but induces costs to avoid (i.e., time) that compete with costs to approach (i.e., pain), and allows for simultaneous assessment of behavioural, psychological, and physiological components. The main aim of the study was to evaluate the new paradigm for its further application. Therefore, we hypothesized that a contingent association between specific approach movements and pain stimuli leads to reductions in these movements, while such approach movements increase again when this association is removed. We further expected changes in self-rated fear of movement-related pain and pain expectancy as well as changes in physiological arousal.

## Methods

### Preregistration

Although the complete research project was publicly available at the Open Science Framework from the start (see: https://osf.io/zh3ak/) and all following studies and Master theses were also formally preregistered (see: https://osf.io/zh3ak/wiki/home/), it was not possible to preregister the current study in any detail due to its explorative character and its primary aim of establishing a new experimental paradigm.

### Participants

The current study includes data from 43 healthy adult participants. The sample size was determined by a-priori power calculations for within-subjects designs (G*Power^28^). Specifically, we intended to detect effect sizes of Cohen’s *d* ~ 0.4 (based on previous work with *N* ranging between 10 and 60) with a statistical power of 80% and an alpha level of 0.05. This resulted in a required sample size of 41 participants. Prior to participation, the following exclusion criteria were ruled out via telephone interviews: Age < 18 or > 35; left-handedness; BMI > 30 or < 18; Psychology student (beyond the 2nd semester); vision and hearing disabilities; insufficient German language skills (to ensure that instructions are understood); regular intake of medication affecting the central nervous system; frequent smoking behaviour (> 15 cigarettes per day); drug consumption (including cannabis); history of or acute diagnosis of chronic pain (e.g., headache); episode of severe pain that required medical treatment within the last six month (e.g., due to an injury); diagnosis of attention-deficit/hyperactivity disorder (ADHD), dyslexia or dyscalculia; acute suffering from any psychological, neurological, cardiovascular, respiratory or endocrinological diseases; disposition for vertigo or fainting. Based on these criteria, the initial pool of more than 500 participants, which were recruited via local (flyer) and online advertisement (e.g., social media, university platforms), was first reduced via e-mail contact to 132 potential candidates with whom telephone interviews were conducted, and finally further reduced to 43. Note that most of the above listed exclusion criteria were determined by the fact that the current study is part of a larger project. This larger project additionally includes a stress induction procedure, the assessment of several physiological (e.g., cortisol) and psychological (e.g., attentional control) variables, and electroencephalographical recordings (EEG) that can be affected by the respective factors in an uncontrollable way (e.g., cortisol levels influenced by smoking, drugs, and medications; attentional control by ADHD diagnosis; EEG by left-handedness). Furthermore, we excluded participants with any history of chronic pain to prevent any re-activation and harm to a potentially hypersensitive nociceptive system.

All participants were asked to abstain from alcohol for 12 h before testing, to not drink any coffee two hours prior to measurement, and to contact the study coordinator directly if they had to take any pain medication on the day of testing (to postpone the measurement to a different date or to exclude the participant in case the intake of pain medication was to become regular). All experimental procedures were in accordance with the declaration of Helsinki and approved by the local ethics committee (Psychological Institute, University of Würzburg, Germany, GZEK 2020-18). Informed written consent according to the declaration of Helsinki and to the guidelines of the German Psychological Society was obtained from all participants. Study participation was reimbursed with 17.50€. One participant was excluded afterwards due to technical problems with data storage. Thus, our final sample included 42 healthy adult participants (21 women; age: *M* = 24.5, *SD* = 3.5, range 19–34).

### Experimental paradigm

**Figure 1:**
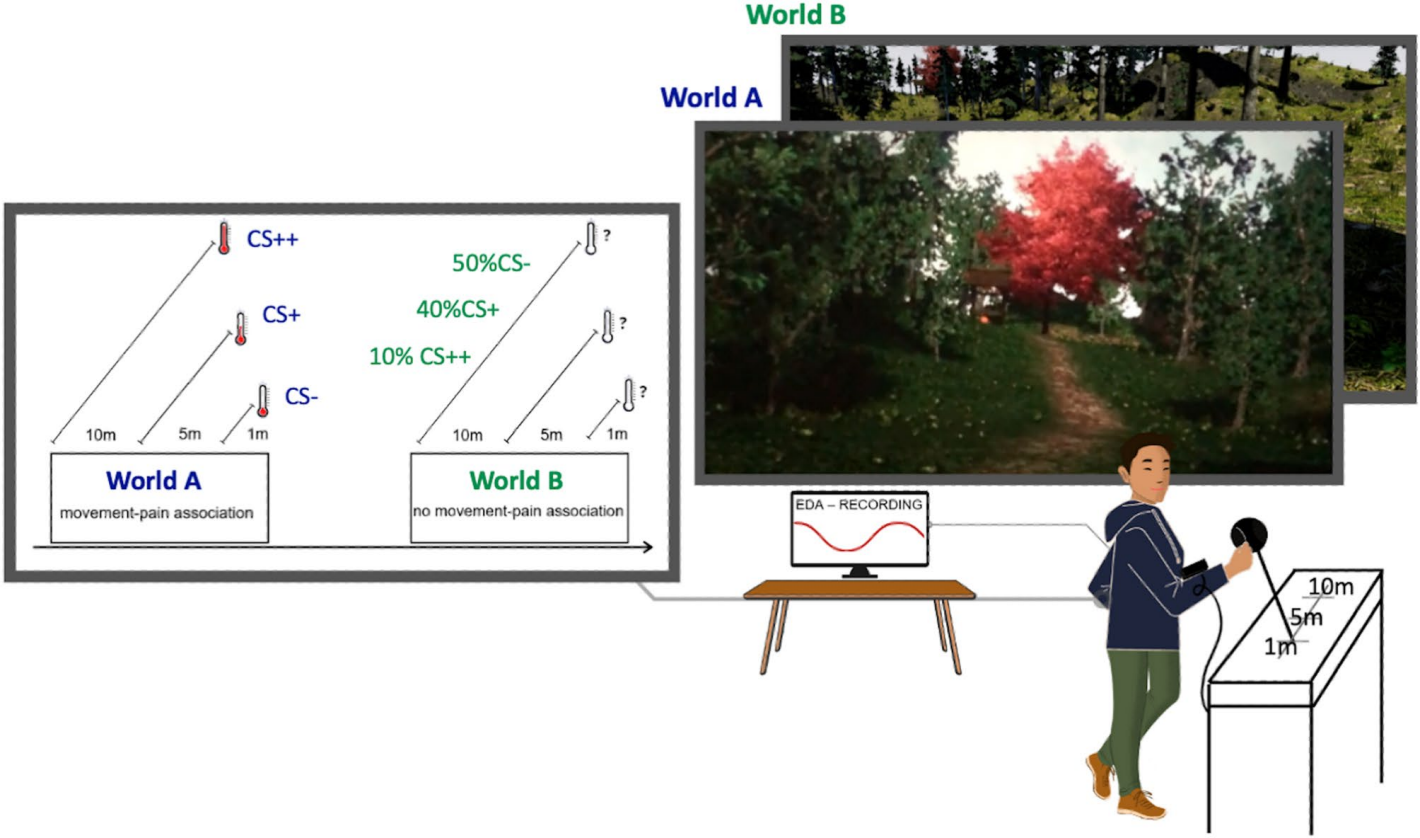
Schematic illustration of the experimental paradigm. In addition to the visual details of both worlds (right), acquisition (world A) and modification phase (world B) also differed in their reinforcement plans (left). While there existed a contingent association between certain movements and certain pain stimuli in world A (acquisition phase), no such association was present in world B (modification phase). Specifically, in world A 1 m steps induced a neutral heat stimulus (CS−), 5 m steps a medium heat pain stimulus (individual heat pain tolerance minus 3 °C, CS+), and 10 m steps were reinforced with a severe heat pain stimulus (individual heat pain tolerance, CS++). In world B, all movements were reinforced according to a fixed and a priori defined reinforcement plan, which was the same for all participants and ensured via pseudo-randomization that 50% of the applied stimuli were neutral stimuli, 40% were medium heat pain stimuli, and 10% were severe heat pain stimuli (see “Methods”). All movements were induced via moving a large joystick apparatus with the right arm that was also subject of the heat pain application, while electrodermal recordings were assessed from the left inner hand.

**Figure 2:**
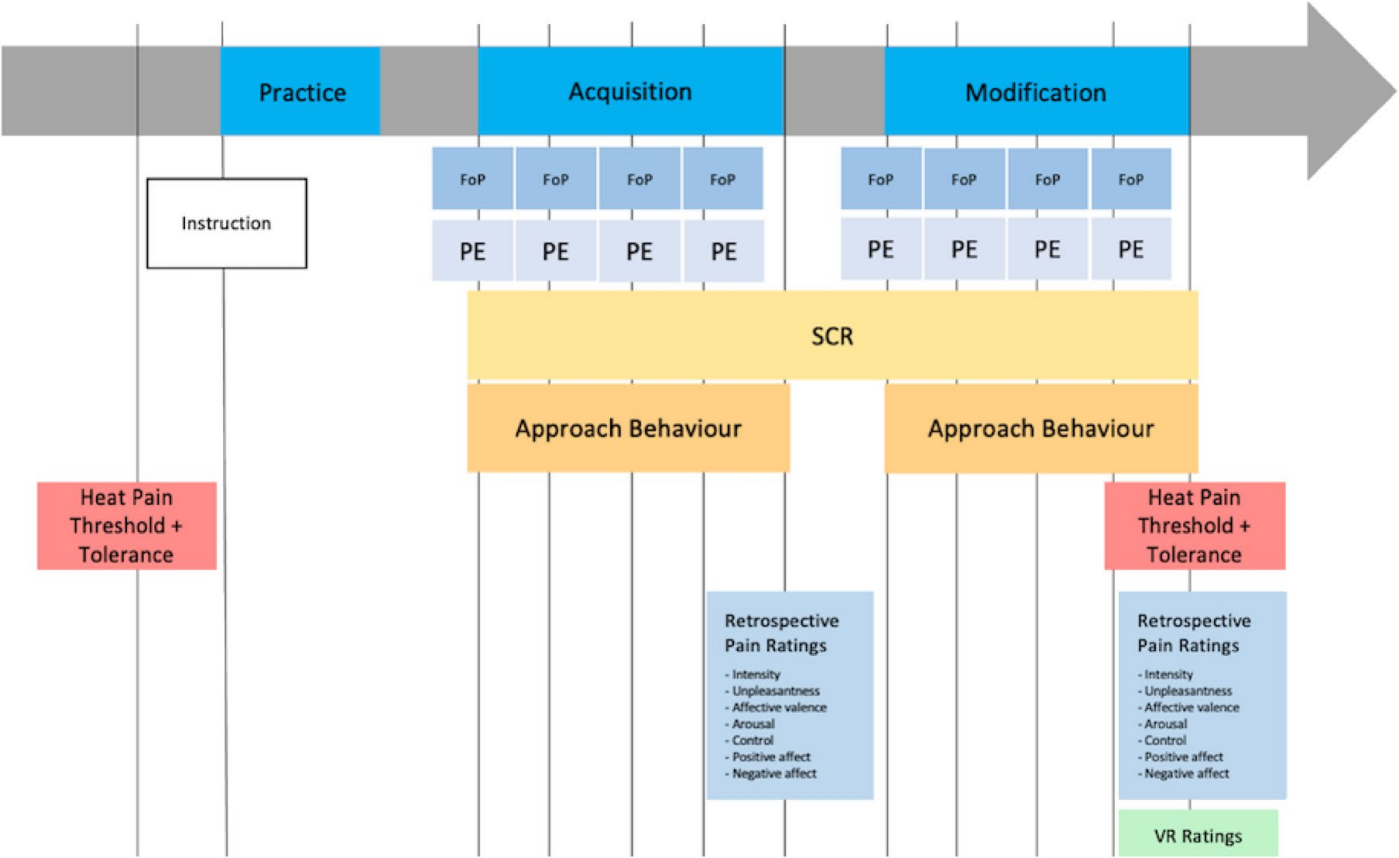
Flowchart of assessed measurements. Heat pain thresholds and heat pain tolerance were assessed in the beginning and at the end of the experiment. Prospective fear of movement-related pain (FoP) and pain expectancy (PE) ratings were obtained before each trial via numerical rating scales (see “Methods”). Additional measures were retrospectively assessed twice, i.e., once after each world: (i) retrospective pain intensity (NRS, 1 = “not painful” to 100 = “very painful”), (ii) retrospective pain unpleasantness (NRS, 1 = “not unpleasant” to 100 = “very unpleasant”, (iii) affective valence (Self-Assessment Manikin Scale, SAM, Bradley and Lang, 1994, nine item version with anchors “happy” and “unhappy”, (iv) arousal (SAM, nine item version with anchors “excited” and “calm”), perceived control (SAM, nine item version with anchors “controlled” and “in control”), (v) positive affect (PANAS, Positive and Negative Affect Schedule, 10 items rated from 1 = “not at all” to 5 = “very strong”), and (vi) negative affect (10 items rated from 1 = “not at all” to 5 = “very strong”, PANAS). Finally, subjective experiences of the VR environment (VR ratings) were assessed with the IPQ (Igroup Presence Questionnaire, 14 Items) and potential feelings of sickness due to the VR were ruled out with the SSQ (Simulator Sickness Questionnaire; 16 Items). Skin conductance levels (SCL) and skin conductance responses (SCR) were assessed during both experimental phases.

The current study follows a within-subject design comprising five phases: A preparation phase, a practice phase, two experimental phases (acquisition, modification), and a postprocessing phase. All phases were passed by all participants in identical order. The paradigm is illustrated in detail in Fig. 1, while Fig. 2 provides a flowchart of acquired measurements. A naturalistic environment was implemented in VR by using a Powerwall, i.e., a computer-created virtual world that was projected on a 2D wallscreen (2 m × 3.22 m) and could be perceived in 3D by wearing customary 3Dglasses (like in the 3D cinema). In this virtual world, participants were instructed to walk along a pre-defined 100 m forest path until they reach the end of the path which could be seen from the starting position. As multiple paths have to be passed, these paths represent the (learning) trials in our paradigm. To manage each trial, participants were advised to use an extended self-made joystick apparatus which allows them to induce three types of virtual steps (1 m, 5 m, 10 m) by different arm movements (small ca. 10 cm, medium ca. 30 cm, large ca. 50 cm) upon which they could freely chose (no further instructions were provided). During these movements, participants stood upright and wore a thermode (see next paragraph) on their lower inner right forearm. Two electrodes for electrodermal recordings were placed at the inner left hand.

The aim of the practice phase was to collect experience with moving in the VR environment. Thus, no electrodermal recordings and no application of heat pain stimuli were applied here. The two experimental conditions (acquisition vs. modification phase) were presented in two distinct virtual environments (acquisition took always place in world A, modification was always realized in world B) that differed with respect to multiple visual characteristics (e.g., type of trees, colour of meadow; for a realistic visualization see Fig. 1), and most importantly, with respect to the reinforcement plan of the thermal stimulation. Those two phases were undergone by all participants in the same order, first world A for acquisition and then world B for modification. While there was a contingent association between certain movements and certain heat stimuli in the acquisition phase (world A: acquisition of the movement-pain association), no such association was present in the modification phase (world B: modification of the movement-pain association). Specifically, in the acquisition phase (world A) small arm movements (inducing a 1 m step in VR) were reinforced with a neutral heat stimulus (CS−, individual pain tolerance minus 10° Celsius), medium arm movements (inducing a 5 m step in VR) were reinforced with a medium heat pain stimulus (CS+, individual pain tolerance − 3 °C), and large arm movements (inducing a 10 m step in VR) were reinforced with a severe heat pain stimulus (CS++, individual pain tolerance). The suitability of exactly these temperatures as well as the absence of any significant differences in “liking” of both worlds and in the preference for “spending time in this environment” were indicated by previous piloting of our experimental setup and by obtaining external ratings (*N* = 19).

The reinforcement rate was 100% in all three cases. In contrast, in the modification phase (world B), the reinforcement with heat stimuli followed a fix plan which was independent of the actually initiated movements and was the same for all participants. This plan was pseudo-randomized by ensuring that applied stimuli included 50% neutral heat stimuli, 40% medium heat pain stimuli, and 10% severe heat pain stimuli (all defined in accordance with the individual pain tolerance measured during the acquisition phase, see above). Note that we selected this approach in contrast to no pain application in the modification phase based on the consideration that also in reality pain is rarely an all or none phenomenon. Recovery from chronic pain (and reductions of pain-related fear) might be characterized by relapses (e.g., reoccurring pain to a movement which was already painless) so that treatments should be robust against these instances of rare but sudden re-occurring pain perceptions. Note further, that all CS+ were above individuals pain thresholds and all CS− were below individual pain thresholds. The heating (and cooling) time of the thermode was the same for all participants and determined by the smallest technically realizable time, i.e., 600 ms for CS−, 2 s for CS+ and 2600 ms for CS++. The heating of the thermode ended as soon as the target temperature was reached, and immediately after, the cooling down started, i.e., there was no delay where the target temperature was held constant. Response costs to reduced approach behaviour were introduced in the form of time required to overcome the eight paths. Specifically, dependent on the amount of approach behaviour, the completion of the acquisition and modification phase could vary between 4 min 48 s (4 × 1 min 12 s when taking only 10 m steps) and 48 min (4 × 12 min when taking only 1 m steps) each. Note that no additional costs to the CS− were implemented as previous piloting indicated that already the benefit in time (walking faster in VR with 10 m steps) led participants to choose this option relatively frequently.

### Study procedure

At the beginning of the experiment, participants were informed about the study details and the fact that they could quit their participation at any time without any disadvantages. Written informed consent was obtained from all participants. Next, participants were asked to complete a demographic questionnaire and the exclusion criteria were checked again. Then, a Peltier thermode (Somedic MSA thermal stimulator; Somedic Sales B, Hörby, Sweden) was fixated on the participants' left forearm and individual heat pain threshold and heat pain tolerance were determined strictly following the procedure of Horn^29^. Note that the maximal applicable temperature was automatically limited by the technical system to 49 °C (to prevent harmful skin irritations) so that potential pain thresholds/tolerances above 49 °C could not be recorded.

Participants entered a light-shadowed room and began with the VR experiment (practice phase). All further details about the experimental procedure were provided to the participants via standardized audio instructions and written projections on the wallscreen. The practice phase consisted of one 100 m path representing one learning trial, while each of the experimental phases comprised four similar trials (4 × 100 m). Specifically, the environment of world A was used in the practice phase and in all trials of the acquisition phase, while the environment of world B was used in all trials of the modification phase. In the practice phase, participants were instructed to try out each type of movement (1 m, 5 m, 10 m step) at least once and to train the virtual walking procedure. Participants waited for a short beep tone before they began with the first joystick movement. Also, they were instructed to rest the joystick for at least one second in one of the three defined target positions to induce a VR movement. The three target positions were highlighted with different colours and luminous stickers on the joystick apparatus. After inducing a step, the joystick was placed back in the original position. Participants were instructed to start with the next movement when the next beep tone occurred after a short delay of six seconds. This delay was implemented to ensure that the thermode could cool down completely after each applied thermal stimulation. Although no thermal stimulation was realized in the practice phase, the general procedure and timing were the same in all conditions (practice phase, acquisition phase, modification phase). After finishing the practice phase, the light was switched on and the thermode as well as the electrodes for electrodermal recordings were placed on the participant’s right arm and left hand, respectively. The light was turned off again and the experiment started with the acquisition phase (world A). During both experimental conditions, online rating formats were presented before each of the four trials on the wallscreen (for an overview of measurements see Fig. 2). These were answered via moving a computer mouse placed on the joystick apparatus.

After completing all three conditions (one trial in the practice phase, four world A trials in the acquisition phase, four world B trials in the modification phase), additional measures were obtained in paper–pencil format (see below and Fig. 2). Also, pain thresholds and tolerances were assessed again to rule out potential effects of habituation or sensitization from the beginning to the end of the experiment. Finally, participants were debriefed about the real study subject (cover story: investigation of pain perception in nature) and reimbursed for their participation. Note that as participants were free to choose which movements they executed to complete the nine trials in VR (one trial in the practice phase, four trials in the acquisition phase, four trials in the modification phase), and by extension, the duration of the experiment varies. On average, the whole testing procedure took 1:45 h. Finally, note that participants wore medical masks during all assessments as data acquisition took place during the COVID-19 pandemic (summer and autumn 2020, Germany).

### Pain-associated approach behaviour and ratings

As main outcome measure approximating pain-associated approach behaviour, the total number of 10 m steps taken to overcome the 100 m distance was assessed for each learning trial (each 100 m path). We focused on 10 m steps as those were linked to the most severe pain stimuli during acquisition and therefore considered as behaviourally most relevant. However, for manipulation check and explorative analyses, the number of 1 m and 5 m steps was also evaluated.

Participants self-reported prospective fear of movement-related pain and prospective pain expectancy for each of the three types of movements (1 m, 5 m, 10 m) was assessed before each learning trial (4 times in each world, Fig. 2). More specifically, participants were asked via standardized audio instructions (i) to which extent they experience fear when they think of performing the movements to cross the next path (fear of movement-related pain, aggregated across all movements of the same type of the next trial) and (ii) to which extent they expect the movements to cross the next path to be painful (pain expectancy, aggregated across all movements of the same type of the next trial). Both answers were assessed online (in the VR) via moving a computer mouse across a numerical rating scale (NRS) with the anchors 1 = “not at all” to 100 = “very strong”. Note that we did not ask participants how they experience fear of pain/pain expectancy in respect to the three types of heat pain stimuli separately to avoid making them aware of these differences and to prevent putting their attention to the underlying contingencies.

Beyond these primary outcome measures, multiple additional measures were obtained to check the experimental manipulation and to explore the new paradigm. These measures were assessed twice, i.e., once after each world and include (Fig. 2): (i) retrospective pain intensity (NRS, 1 = “not painful” to 100 = “very painful”), (ii) retrospective pain unpleasantness (NRS, 1 = “not unpleasant” to 100 = “very unpleasant”, (iii) affective valence (Self-Assessment Manikin Scale, SAM^30^, nine item version with anchors “happy” and “unhappy”, high internal consistency: *α* = 0.83–0.89; acceptable retest-reliability *r*_tt_ = 0.55–0.78 in Ref.^31^), (iv) arousal (SAM, nine item version with anchors “excited” and “calm”), perceived control (SAM, nine item version with anchors “controlled” and “in control”), (v) positive affect (PANAS, Positive and Negative Affect Schedule^32^, 10 items rated from 1 = “not at all” to 5 = “very strong”, German adaption: Ref.^33^; high internal consistency: *α* ≥ 0.84 but rather low retest-reliability *r*_tt_ ≤ 0.66), and (vi) negative affect (10 items rated from 1 = “not at all” to 5 = “very strong”, PANAS). Finally, the subjective experience of the VR environment was assessed with the IPQ (Igroup Presence Questionnaire, 14 Items^34^; high internal consistency: *α* = 0.87; sufficient retest-reliability *r*_tt_ = 0.74 in Ref.^35^) and potential feelings of sickness due to the VR were ruled out with the SSQ (Simulator Sickness Questionnaire; 16 Items^36^; acceptable internal consistency: *α* ≥ 0.70; low retest-reliability *r*_tt_ ≤ 0.73). The latter two measures (assessing VR experience) were only assessed once, after finishing the whole VR experiment (results not reported here). All measures were used in the German-adapted versions or translated into German.

### Electrodermal recordings and preprocessing

Electrodermal activity was recorded with a sample rate of 1000 Hz during both experimental conditions with Brain Vision Recorder (Brain Products GmbH, München) and a V-Amp Amplifier (Brain Products GmbH, München^37^). Therefore, two passive Ag/AgCl surface electrodes (filled with 0.05 molar NaCl electrode paste) were placed on the participants' non-dominant (left) hands' thenar and hypothenar. Two measures were calculated for each experimental trial: Skin conductance levels (SCL) as tonic indicator of physiological arousal and skin conductance responses (SCR) following 10 m steps as phasic behaviour-related arousal measure, i.e., directly linked to the pain-associated movement (CS++).

Data preprocessing was conducted with Brain Vision Analyser 2.0 (Brain Products GmbH, München). After raw data were band-pass filtered between 1 and 45 Hz, data segments were defined from 1000 ms before to 8000 ms after movement induction (i.e., the timepoint when participants' arm reached its target position, and the VR step was initiated). SCRs were calculated as the difference between voltage minima and maxima occurring between 800–4000 ms and 2000–8000 ms, respectively, as responses in skin conductance are assumed to take place within this time frame^38,39^. Automatically determined extrema were manually controlled (removed or changed if required). Bad segments (i.e., maximum occurred before the minimum) were marked with − 1 to exclude such segments from further analyses. Finally, all valid SCRs were averaged across segments. This leaves the three SCR measures (electrodermal responses to 1 m, 5 m, 10 m steps) for each of the eight experimental learning trials (four 100 m paths in world A, four 100 m paths in world B, Fig. 2). Trial-wise SCLs were calculated as mean electrodermal activity within the time frame of each experimental trial, thus providing eight SCL values per subject.

### Statistical analyses

First, multiple descriptive statistics were performed to check whether our experimental manipulation was successful. Mean values of measures (e.g., to check if pain tolerances were lower than pain thresholds) were compared using paired *t*-tests in cases in which the Shapiro–Wilk test indicated no significant deviations from normality. Otherwise, the Wilcoxon signed-rank test was used instead. The *t*-tests' effect sizes were estimated using Cohen’s *d*, while for Wilcoxon signed-rank tests the matched rank biserial correlation *r* was used. To assess the primary outcome measures for our main hypotheses we tested for main effects of the factors learning Trial (1–4) and World (A, acquisition phase vs. B, modification phase) as well as for their interaction via two-factorial repeated-measures analyses of variance (rm-ANOVAs). The Greenhouse–Geisser adjustment was applied when the assumption of sphericity was violated (as indicated by the Mauchly test if *p* < 0.05). Although the factor World has only two levels (A,B), we followed the recent statistical recommendations and refrained from using a non-parametric alternative or additional data transformations as ANOVA has been demonstrated to be sufficiently robust against potential variations in measurement scales^40,41^. Effect sizes for rm-ANOVAs were reported using partial Eta squared *η*^2^*p*. For skin conductance responses participants were excluded from statistical analyses when no responses could be recorded in the conditions of interest. This affected eight participants. Note that in this proof-of-concept study we performed no further outlier exclusion as we aimed to explore all possible behavioural response patterns to our new experimental paradigm. These decisions were made a priori. Statistical analyses were conducted in R (version 1.3.1056, www.r-project.org), the R-based software packages JASP (version 0.13.1, www.jasp-stats.org), and the R-based program jamovi (version 1.2; the jamovi project; www.jamovi.org). Statistical significance was accepted at* p* < 0.05. Note that the global results concerning our five main outcome variables (i.e., rm-ANOVAS of pain-associated approach behaviour, fear of movement-related pain and pain expectancy ratings, SCR, and SCL) are reported (a) uncorrected for multiple comparisons for exploratory purposes, and (b) corrected for multiple (five) comparisons with Bonferroni (adjusted *p* < 0.01). Results of post-hoc *t*-tests of main outcome variables were also reported (a) uncorrected, and (b) corrected for multiple (four) trial-wise comparisons with Bonferroni (adjusted *p* < 0.0125). Manipulation checks were only reported uncorrected.

## Results

### Experimental manipulation

Individual baseline pain thresholds varied between 35.4 °C and 49 °C (*M* = 42.8, *SD* = 4.2) and were significantly lower than baseline pain tolerances (38.8 °C to 49 °C; *M* = 47.1, *SD* = 2.6; *t*(41) = − 9.909, *p* < 0.001, Cohens *d* = − 1.529). Pain thresholds assessed post-experimentally varied between 35.3 °C and 48.9 °C (*M* = 42.4, *SD* = 4.1) and were also significantly lower than the respective pain tolerances (40.5 °C to 49 °C; *M* = 47.4, *SD* = 2.4; *t*(41) = − 11.156, *p* < 0.001, Cohens *d* = − 1.721; see also Table 1). Of note, neither pain thresholds nor pain tolerances changed significantly from pre to post experimental assessments (pain threshold: *t*(41) = 1.399, *p* = 0.169, Cohens *d* = 0.216; pain tolerance: *W*(41) = 129.0, *p* = 0.796, *r* = − 0.065). Together, these results support the validity of the individual pain assessments and speak against obvious effects of sensitization or habituation over the course of the experiment.

**Table 1:** Descriptive statistics of manipulation check variables, explorative and primary outcome measures.

	M (SD)	Min	Max	M (SD)	Min	Max
**Manipulation checks and exploratively assessed variables**						
	Before experiment			After experiment		
Pain threshold	42.8 (4.2)	35.4	49	42.4 (4.1)	35.3	48.9
Pain tolerance	47.1 (2.6)	38.8	49	47.4 (2.4)	40.5	49
	After acquisition phase (World A)			After modification phase (World B)		
Positive affect (PANAS)	28.8 (6.3)	17	44	28.3 (8.0)	14	42
Negative affect (PANAS)	13.1 (3.6)	10	23	11.7 (2.1)	10	19
Pain intensity	49.4 (27.8)	0	89	31.3 (20.4)	0	66
Pain unpleasantness	56.5 (28.8)	0	100	36.1 (22.9)	0	71
Happiness/unhappiness	5.7 (1.4)	3	9	6.3 (1.8)	2.3	10
Arousal	3.7 (2.1)	1	8	3.3 (2.5)	0	8.6
Control	7.2 (1.8)	1	9	6.1 (3.1)	1.2	10
**Primary outcome measures**						
	During acquisition phase (World A)			During modification phase (World B)		
Number of 10 m steps	23.8 (11.2)	2	40	34.9 (5.0)	23	40
Fear of movement-related pain	4.3 (4.7)	0	20.5	3.9 (4.6)	0	19.8
Pain expectancy	16.5 (10.1)	1	35.5	13.8 (9.0)	0	40.0
SCL	12.1 (4.9)	3.4	25.9	12.6 (4.8)	3.8	25.4
SCR to 10 m Steps	1.2 (0.8)	0.1	4.6	1.0 (0.6)	0.2	3.7

**Figure 3:**
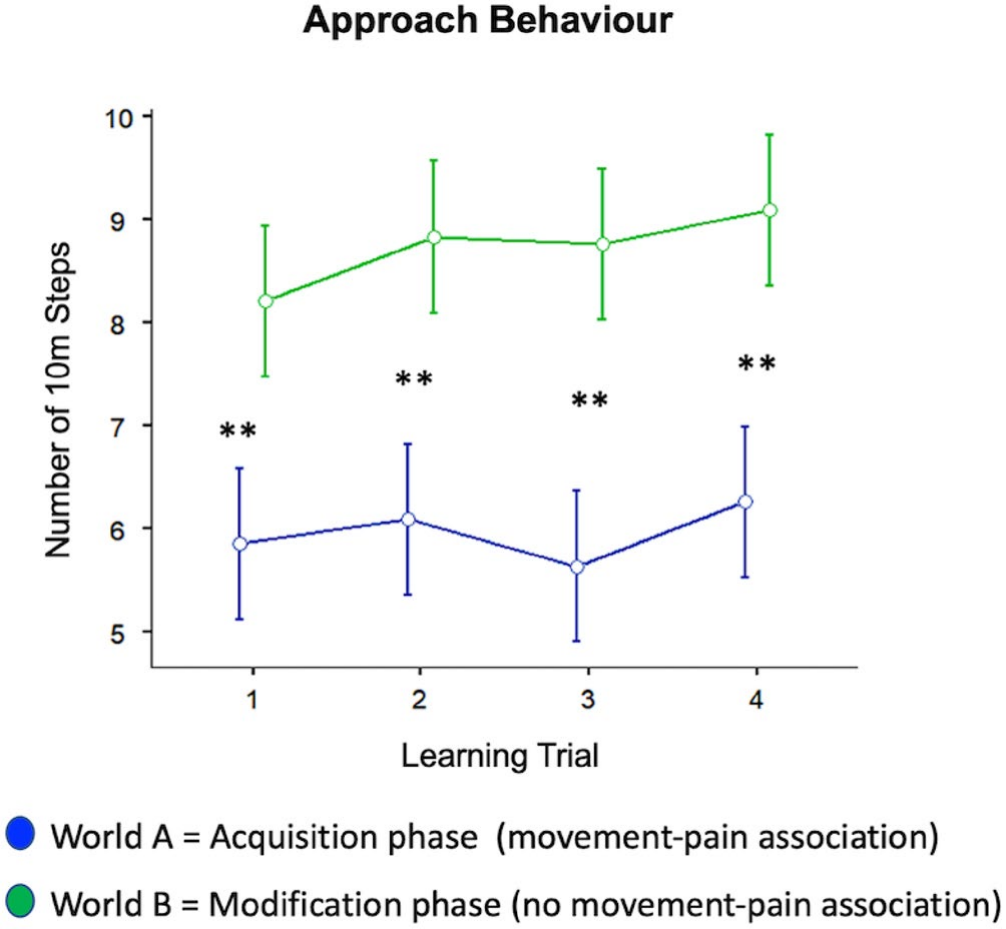
Pain-associated approach behaviour (total number of 10 m steps) over the four learning trials in the acquisition phase (world A, contingent movement-pain association) and in the modification phases (world B, no contingent movement-pain association). The graphs illustrate the two-factorial repeated-measures ANOVAs conducted as main analyses with factors learning Trial and World (acquisition vs. modification phase) (see Results section for further details). Error bars indicate 95% confidence intervals. **p < 0.01 indicating statistical significance of post-hoc paired *t*-tests (uncorrected for multiple comparisons), i.e., directly comparing two learning trials between both phases (acquisition vs. modification phase).

**Figure 4:**
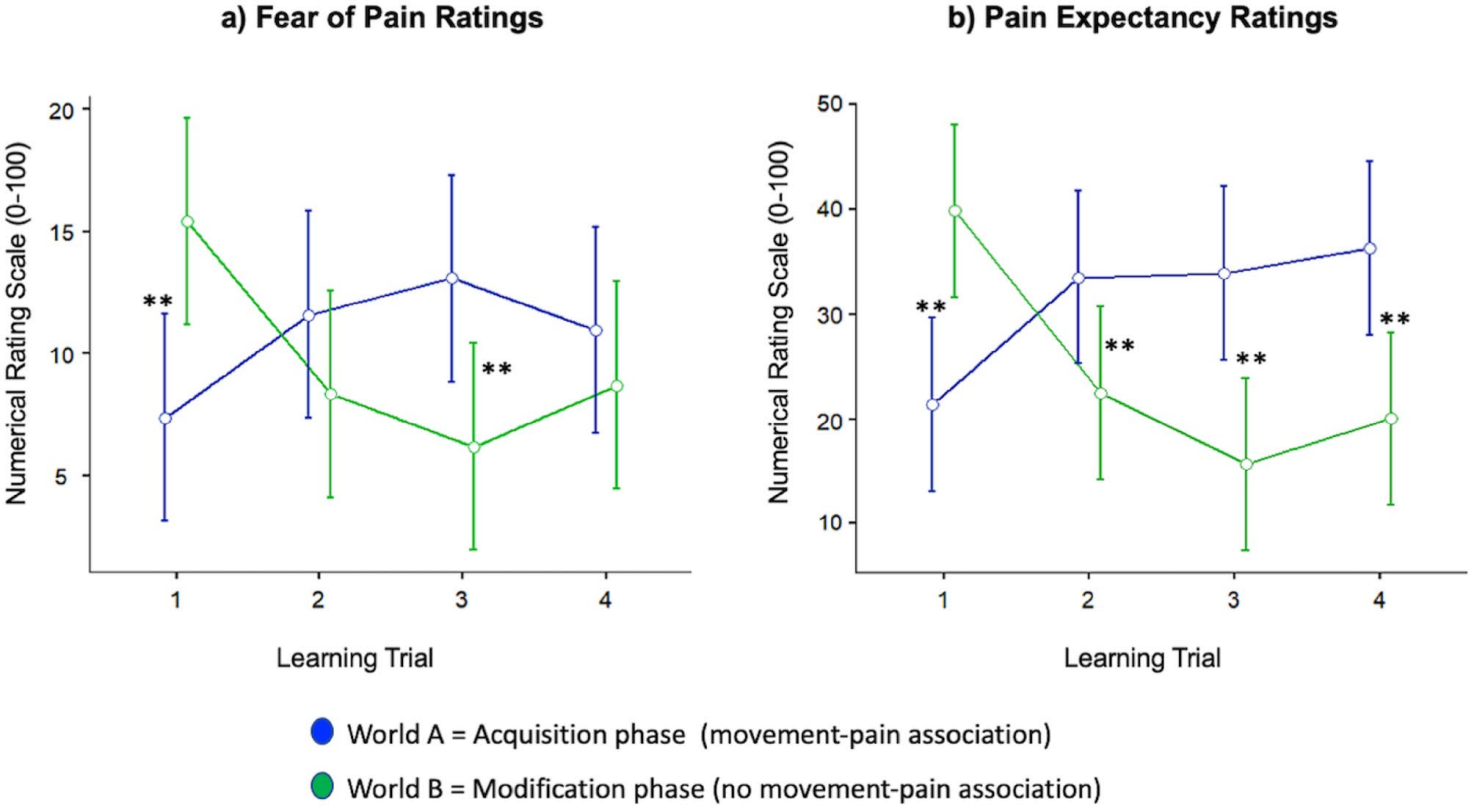
Fear of movement-related pain (**a**) and pain expectancy (**b**) ratings over the four learning trials in the acquisition phase (world A, contingent movement-pain association) and in the modification phases (world B, no contingent movement-pain association). The graphs illustrate the two-factorial repeated-measures ANOVAs conducted as main analyses with the factors learning Trial and World (acquisition vs. modification phase) (see “Results” section for further details). Error bars indicate 95% confidence intervals. Ratings were assessed on a numerical rating scale (NRS) ranging from 1 to 100. **p < 0.01 indicating statistical significance of post-hoc paired *t*-tests (uncorrected for multiple comparisons), i.e., directly comparing two learning trials between both phases (acquisition vs. modification phase).

**Figure 5:**
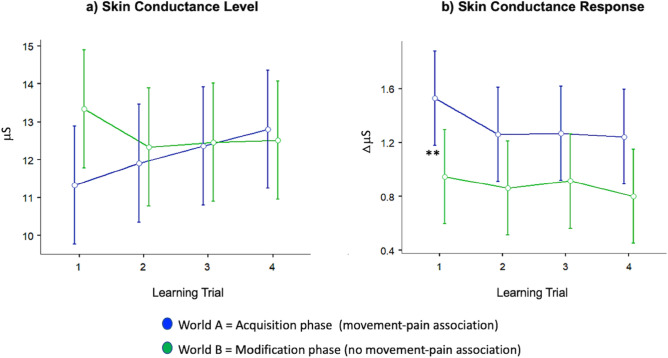
Skin conductance levels (**a**) and skin conductance responses (**b**) over the four learning trials in the acquisition phase (world A, contingent movement-pain association) and in the modification phases (world B, no contingent movement-pain association). The graphs illustrate the two-factorial repeated-measures ANOVAs conducted as main analyses with factors learning Trial and World (acquisition vs. modification phase) (see “Results” section for further details). Skin conductance levels (SCL) and skin conductance responses (SCR) are depicted in µS (micro Siemens). Error bars indicate 95% confidence intervals. **p < 0.01 indicating statistical significance of post-hoc paired *t*-tests (uncorrected for multiple comparisons), i.e., directly comparing two learning trials between both phases (acquisition vs. modification phase).

Pain threshold and pain tolerances are depicted in °C. The maximal applicable temperature was 49 °C (technically determined to prevent skin irritations) so that potential pain thresholds/tolerances above 49 °C could not be recorded. Positive and negative affect were rated with 10 items from 1 = “not at all” to 5 = “very strong” so that obtained sum values could vary between 10 and 50. Pain intensity and pain unpleasantness were rated on a NRS from 1 to 100, while happiness/unhappiness, arousal, and control were assessed with the Self-Assessment Manikin Scale (SAM; 9 item version with anchors “happy” and “unhappy). Note that the 9 visual categories were translated into values ranging from 0 to 10 for statistical analyses. Fear of movement-related pain and pain expectancy were rated on a NRS from 1 to 100. Skin conductance responses (SCR) and skin conductance levels (SCL) are depicted in µS (micro Siemens). Primary outcome measures were averaged across trials. Trial-specific primary outcome measures are depicted in Figs. 3, 4 and 5. For additional exploratorily assessed variables (number of 1 m and 5 m steps, SCR to 1 m and 5 m steps) see Supplementary Table S1. *PANAS* Positive and negative affect schedule.

Positive and negative affect ratings as assessed with the PANAS are depicted in Table 1. While positive affect was similar after the acquisition phase (world A) and after the modification phase (world B) (*t*(41) = 0.921, *p* = 0.362, Cohens *d* = 0.142), negative affect was significantly higher after the acquisition phase compared to after the modification phase (*W*(41) = 252.5, *p* < 0.001, *r* = 0.830). Pain intensity and pain unpleasantness ratings also decreased significantly from after the acquisition phase to after the modification phase (PI: *t*(41) = 5.088, *p* < 0.001, Cohens *d* = 0.785; PU: *t*(41) = 6.074, *p* < 0.001, Cohens *d* = 0.866; Table 1). In sum, these findings suggest that negative affect, pain intensity, and pain unpleasantness can be modified by a change in movement-pain contingencies, while positive affect seems to be rather unaffected (for results of additional variables registered to evaluate the experimental manipulation see Supplement).

Finally, in the acquisition phase skin conductance responses (SCRs) to 1 m steps (paired with neutral heat stimuli) were significantly lower than SCRs to 5 m steps (paired with medium heat pain stimuli) (*W*(41) = 29.0, *p* < 0.001, *r* = − 0.875), and SCRs to 10 m steps (paired with severe heat pain stimuli) were significantly higher than SCRs to 5 m steps (paired with medium heat pain stimuli) (*W*(41) = 75.0, *p* < 0.001, *r* = − 0.834). These physiological responses support the assumption that different pain-movement associations trigger different levels of arousal dependent on the strength of the pain stimulus.

### Main outcome variables

The main aim of the current study was to test whether the developed experimental paradigm can be used to comprehensively investigate the acquisition and modification of pain-associated approach behaviour, as well as pain-related psychological and physiological factors. Specifically, we hypothesized that pain-associated approach behaviour decreases in world A (acquisition phase) where a contingent movement-pain association exists, while it increases when this association is removed (world B, modification phase). We further expected accompanying changes in self-rated fear of movement-related pain, in pain expectancy, and in physiological arousal, i.e., increase over the trials in world A and decrease over the trials in world B.

#### Pain-associated approach behaviour

Figure 3 illustrates the changes in pain-associated approach behaviour in response to our experimental manipulation. Pain-associated approach behaviour was operationalized as number of 10 m steps as those were paired with the most severe heat pain stimuli (CS++) during the acquisition phase. No significant interaction effect was observed (F(2.05,83.92) = 1.54, *p* = 0.220, *η*^2^*p* = 0.036), but a significant main effects of World (F(1,41) = 51.72, *p* < 0.001, *η*^2^*p* = 0.558) and Trial (F(2.53,103.73) = 5.53, *p* = 0.003, *η*^2^*p* = 0.119). Both effects stay also significant when correcting for the number of main outcome variables (five, see “Methods”) with Bonferroni (adjusted *p* < 0.01). Post-hoc *t*-tests (uncorrected for multiple comparisons) revealed significant differences in all trials between both phases (see Fig. 3). As all *p* < 0.01, these effects also hold when correcting for the number of trial-wise (four, see “Methods”) comparisons with Bonferroni (adjusted *p* < 0.0125). Together these results suggest reduced pain-associated approach behaviour in the acquisition phase (contingent movement-pain association) compared to the modification phase (no contingent movement-pain association). Further, when averaging across both phases, pain-associated approach behaviour seems to increase slightly over the course of the experiment. Although the increase of approach behaviour in the modification phase was obviously responsible for this main effect of Trial (see Fig. 3), the pattern of changes (as indexed by the interaction) did not differ significantly between both phases. For completeness, results for 5 m and 1 m steps are illustrated in Supplementary Table S1 and Supplementary Fig. S1.

#### Fear of movement-related pain and pain expectancy

Figure 4 depicts the results for the prospective fear of movement-related pain and pain expectancy self-ratings. Concerning fear of movement-related pain, we observed a significant Trial × World interaction (F(2.01,82.47) = 14.164, *p* < 0.001, *η*^2^*p* = 0.257), while no main effects were observed (factor World (F(1,41) = 1.220, *p* = 0.276, *η*^2^*p* = 0.029; factor Trial (F(1.53,62.54) = 0.635, *p* = 0.492, *η*^2^*p* = 0.015). This  interaction effect stays significant when correcting for the number of main outcome variables (five) with Bonferroni (adjusted *p* < 0.01). Post-hoc *t*-tests (uncorrected for multiple comparisons) revealed the existence of two significant trial-specific differences: (1) fear of movement-related pain was lower before trial 1 of the acquisition phase than before trial 1 of the modification phase and (2) fear of movement-related pain was higher before trial 3 of the acquisition phase than before trial 3 of the modification phase (see Fig. 4a). As all *p* < 0.01, these effects also hold when correcting for the number of trial-wise (four) comparisons with Bonferroni (adjusted *p* < 0.0125). These results suggest that the experimentally induced change in the movement-pain contingency between acquisition and modification phase led to significant alterations in prospective ratings of fear of movement-related pain.

Concerning pain expectancy, Fig. 4b illustrates a significant Trial × World interaction (F(1.77,72.38) = 29.00, *p* < 0.001, *η*^2^*p* = 0.414) as well as a significant main effect of World (F(1,41) = 11.35, *p* = 0.002, *η*^2^*p* = 0.217). No main effect was observed for the factor Trial (F(1.76,72.17) = 2.49, *p* = 0.097, *η*^2^*p* = 0.057). The interaction effect remains significant when Bonferroni corrected for the number of main outcome variables (five; adjusted *p* < 0.01). Post-hoc *t*-tests (uncorrected for multiple comparisons) reveal the existence of significant differences for all trial-wise comparisons. While self-rated pain expectancy was lower before trial 1 of the acquisition phase than before trial 1 of the modification phase, pain expectancy was higher before trial 2, 3, and 4 of the acquisition phase than before trial 2, 3, and 4 of the modification phase, respectively (see Fig. 4b). Also, these effects hold when correcting for the number of trial-wise comparisons (four) with Bonferroni (adjusted* p* < 0.0125). These results suggest that the change in contingency between both experimental phases led to significant changes in prospective pain expectancy ratings.

#### Electrodermal activity

Physiological arousal was assessed via skin conductance levels (SCLs, tonic response over the course of each learning trial, Fig. 5a) and skin conductance responses to 10 m steps (SCRs, phasic response, Fig. 5b). Concerning SCL, a significant World × Trial interaction was observed (F(1.37,56.06) = 7.41, *p* = 0.004, *η*^2^*p* = 0.153), while no main effects were present for the factors World (F(1,41) = 1.94, *p* = 0.171, *η*^2^*p* = 0.045) and Trial (F(1.79,73.53) = 1.26, *p* = 0.288, *η*^2^*p* = 0.030). This interaction stays also significant when correcting for the number of main outcome variables (five) with Bonferroni (adjusted *p* < 0.01). Figure 5a suggests that physiological arousal increased during the acquisition phase followed by a reduction during the modification phase, however, post-hoc *t*-tests (uncorrected for multiple comparisons) revealed the absence of any significant difference for all trial-wise comparisons between two phases. Nevertheless, the significant interaction effect suggests that the change in contingency between both experimental phases causes differences in physiological arousal reflected in SCL.

Movement-specific (phasic) changes in physiological arousal were approximated via skin conductance responses to 10 m steps. We observed no World × Trial interaction (F(1.67,55.04) = 0.500, *p* = 0.683, *η*^2^*p* = 0.057) but a significant main effect of World (F(1,33) = 5.248, *p* = 0.028, *η*^2^*p* = 0.137). No main effect of the factor Trial was found (F(1.93,63.78) = 1.419, *p* = 0.242, *η*^2^*p* = 0.041). The main effect of World stays significant when correcting for the number of main outcome variables (five) with Bonferroni (adjusted *p* < 0.01). Post-hoc *t*-tests (uncorrected for multiple comparisons) revealed the existence of one significant difference for trial-wise comparisons that also survives the Bonferroni correction for the number of trial-wise comparisons (adjusted* p* < 0.0125). Specifically, skin conductance response to 10 m steps in trial 1 of the acquisition phase was significantly higher than in trial 1 of the modification phase (see Fig. 5b). Please note that this result pattern resembles the pattern observed for pain-associated approach behaviour. For completeness, results for SCR to 5 m and 1 m steps are illustrated in Supplementary Table S1 and Supplementary Fig. S2.

### Post-hoc control analyses

#### Temporally resolved approach behaviour to pain

The results concerning pain-associated approach behaviour (see above) revealed a significant main effect of World. This effect may be driven (a) by the existence of per-se difference in the amount of approach behaviour between both phases, or (b) by very fast adaptations of approach behaviour within the first trial. To explore both possibilities, we temporally resolved the changes in pain-associated approach behaviour. Specifically, we subdivided the first virtual 100 m path (learning trial) into four 25 m paths (learning subtrials) and analyzed the relative amount of 10 m steps taken within each of these 25 m subtrials (see Supplementary Fig. S3a). Note that we divided the 100 m path into four subtrials as more subdivisions provided a less accurate estimate due to higher numbers of overlapping steps, i.e., steps started in one subtrial but were completed in another.

The two-factorial rm-ANOVA revealed a significant interaction between the factor World and the factor Subtrial (F(3,123) = 6.371, *p* < 0.001, *η*^2^*p* = 0.134) and a significant main effect of the factor World (F(1,41) = 42.222, *p* < 0.001, *η*^2^*p* = 0.505), while no main effect was found for the factor Subtrial (F(2.46,114.39) = 0.376, *p* = 0.731, *η*^2^*p* = 0.009). Both effects also stayed significant when correcting for the number of main outcome variables (five) with Bonferroni (adjusted *p* < 0.01). These results reveal that in addition to the average amount of pain-associated approach behaviour (main analysis, see above) the pattern of changes also varied between both experimental phases. In sum, these findings suggest that pain-associated approach behaviour was reduced and enhanced rapidly during both the acquisition and modification phase (option b).

For additional exploratory insights, the pain-associated approach behaviour of the other three trials was also temporally resolved. The patterns over the respective 16 subtrials are illustrated in Supplementary Fig. S3b. A two-factorial rm-ANOVA revealed a significant interaction effect between the factor World and the factor Subtrial (F(8.48,347.69) = 2.72, *p* = 0.005, *η*^2^*p* = 0.062), a significant main effect of the factor World (F(1,41) = 50.59, *p* < 0.001, *η*^2^*p* = 0.552), and a significant main effect of the factor Subtrial (F(8.33,341.45) = 2.61, *p* = 0.008, *η*^2^*p* = 0.060). All effects stay significant when correcting for the number of main outcome variables (five) with Bonferroni (adjusted *p* < 0.01). Therefore, also these results point towards option b. Approach behaviour is quickly reduced when a contingent movement-pain association exists, while it is also quickly increased when this contingency is removed.

#### Skin conductance response: fear vs. physiological reaction to pain

A further and purely exploratory post-hoc analysis was conducted to evaluate the possibility that significant differences between both phases in SCR (see above and Fig. 5a) may merely reflect differences in the physiological reaction to pain instead of fear of movement-related pain. This would imply that respective changes in SCR could only be a reflection of the different amounts of painful stimuli applied in both worlds (all 10 m steps were paired with CS++ in world A, contingent association, while only some 10 m steps were paired with CS++ in world B, random association). Therefore, we extracted the SCRs from only those 10 m steps in world B that were followed by neural heat stimuli (CS−). Those might reflect the “pure” fear component free of any potential physiological reaction to heat pain. The observed pattern is illustrated in Supplementary Fig. S4. It confirms that the SCR observed during our paradigm reflects physiological arousal different from the physiological reaction to the pain itself (e.g., reflecting fear of movement-related pain and pain expectancy).

## Discussion

Based on empirical background suggesting critical relevance of approach-avoidance behaviour for the development and maintenance of chronic pain, we propose a new Virtual Reality paradigm to investigate the involved learning mechanisms within a controlled laboratory setting. We experimentally manipulated the contingency between three types of arm movements and three types of pain stimuli in a sample of *N* = 42 healthy adults. We observed a fast reduction in pain-associated approach behaviour when a contingent movement-pain association existed, while approach increased immediately when this contingency was removed. Respective contingency changes manifested also in anticipatory self-ratings capturing fear of movement-related pain and pain expectancy, as well as in physiological arousal (i.e., skin conductance). Manipulation checks further indicate the absence of effects of habituation or sensitization over the course of the experiment and that, as intended, different movement-pain associations induce different levels of arousal dependent on the strength of the pain stimulus.

The Fear Avoidance Model of chronic pain proposes that psychological factors are critical in the development and maintenance of chronic musculoskeletal pain. More specifically, it describes how an acute or potential harm can create a self-reinforcing vicious cycle between fear of movement-related pain and pain-related avoidance behaviours^3,4,6^. In the following, a large number of research endeavours addressed associative learning mechanisms involved in the acquisition of fear of movement-related pain. Clever experimental paradigms like the voluntary joystick movement paradigm were developed and adapted extensively^16^. Typically, in such paradigms, a specific movement (e.g., hand movement to the right) represents the conditioned stimulus (CS+) that was former neutral but obtains the potential to elicit the conditioned reaction. Therefore, multiple pairings of the CS+ with an unconditioned stimulus (US, pain) are required. Research based on these paradigms suggests that the acquisition of fear of movement-related pain can be explained by mechanisms of classical conditioning. This also applies to phenomena of (over-) generalization^7,42^, extinction, and return of the fear of movement-related pain^43,44^.

In contrast, reduced approach behaviour and extensive avoidance might be better described by operant learning mechanisms (i.e., instrumental learning; the learning about the avoidance behaviours’ consequences^15^). More specifically, the Fear Avoidance Model assumes that successful avoidance is reinforced by the non-occurrence of the feared pain and the prevented catastrophe of acute harm^4,5^. Such operant mechanisms are especially important when trying to understand the persistence of chronic pain^14^. First experimental paradigms were developed to address these mechanisms in the context of movement-related pain (e.g., robotic arm paradigm^10^). Our proposed VR paradigm builds directly on this research and extends it by combining *associative* *learning* of contingencies between a certain arm movement and a certain pain stimulus with *operant learning* of avoidance behaviour. To realize associative learning, participants were neither instructed to approach or avoid nor informed about the contingency, but had to explore and detect the association completely on their own (without any guidance), most likely via mechanisms of classical conditioning. To realize operant learning of avoidance behaviour, we implemented two types of reinforcement that contradict each other and were thus required to be balanced out. Specifically, pain-associated approach behaviour (10 m virtual steps performed via hand movements to reach a virtual goal at the end of a virtual path) were coupled with heat pain stimuli, while we also implemented costs to pain-associated avoidance behaviour (1 or 5 m steps) in the form of additional time required to overcome the path and to reach the final goal.

As summarized above, we observed specific effects related to the experimental manipulation of the movement-pain (CS-US) contingency in line with our hypotheses. Changes in the behaviour-pain contingency induced changes in pain-associated approach behaviour, fear of movement-related pain and pain expectancy, as well as in physiological arousal.

The following unexpected observations and shortcomings of the study have to be discussed. First, the change in contingency affected behaviour faster than self-ratings. However, please note that self-ratings were assessed for the three types of movements separately. While we discarded more detailed ratings as we anticipated a disruption of VR presence, future studies should consider movement specific assessment of fear of movement and pain expectancy. A second observation for which we have currently no explanation is the absence of significant differences in arousal and perceived control between both experimental phases as well as the significantly reduced negative affect after the modification phase. This is counterintuitive, as participants lost control over which pain stimuli they received in the modification phase (pseudo-random reinforcement). However, at least the participants in this proof-of-concept study selected 10 m steps in the acquisition phase (world A) relatively often, thus that in comparison to the modification phase (world B) in which painful stimuli (CS++ and CS+) were applied in accordance with a fixed reinforcement plan, overall much more pain was applied during acquisition. Specifically, participants received on average a total (sum over all four trials) of 23.9 CS++ stimuli during acquisition, while during modification the average number of applied CS++ was 4.8. Similarly, the average number of applied CS+ stimuli during acquisition was 32, while during modification the average number of CS+ was 19.2. Whether and to which extent the fewer number of CS++ and CS+ compensates for the lack of controllability with respect to negative affect, presents an interesting subject for future investigation. Fourth, the generalizability of our proof-of-concept study is also limited by our strict inclusion criteria and the fact that we prevented heat stimuli above 49 °C due to concerns of the University’s ethic committee, i.e., to really guarantee the absence of any skin burn or harm. In consequence, we cannot ensure that all participants’ heat pain tolerance was actually captured by the CS++ (for 18 out of 43 participants the calibration reached 49 °C). However, please note that this restriction actually worked against our hypothesis so that we conclude that our hypotheses-supporting findings are valid. Nevertheless, future studies should use larger and more representative samples to obtain more comprehensive insights about the robustness of findings and use stimulation methods which allow better capturing each participant’s heat pain tolerance, e.g., with wave-like heat pain stimulation or longer stimuli durations. Fifth and last, the duration of the time spent in the virtual environment, and thus the whole situation of being alert for receiving pain stimuli, could vary between 9 min 36 s (only 10 m steps) and 1 h 36 min (only 1 m steps). Although, our participants were all relatively fast (11 participants selected only 10 m steps and no participant only 1 m steps) this variation in time hinders not only the comparison of outcome variables between participants in our study but refers also to cross-study comparisons in general (for completeness note that the mean duration of the whole experimental procedure implying the whole setup, instructions, questionnaires, and all three experimental phases was 144 min with a standard deviation of 17 min).

In the following section we outline methodological aspects that require further consideration and suggest promising future adaptations of our paradigm. At first, the self-ratings created to capture fear of movement-related pain were generally low, likely because our participants referenced the German word “Angst” to a very strong inner feeling of threat (e.g., to have an accident). Although, there was still enough variance to observe the respective effects, the framing of the question should be adapted in future studies. Second, all results concerning SCR should be interpreted with caution and as hypotheses generating as we implemented a rather conservative censoring to the SCR by excluding (instead of replacing) all trials with missing values. This led to an exclusion of up to eight participants in the SCR analysis. Future studies might therefore explore whether different censoring methods come to similar effects. Third, the pairing between phases and virtual environments was always the same in the current study, i.e., the acquisition phase always took place in world A, while the modification phase was always realized in world B. Although we consider it as relative unlikely that acquisition or modification works better or worse in the one of the two environments, future research could easily randomize this pairing to exclude any remaining doubts.

Fourth, to optimally investigate how a change in CS-US contingencies manifests in multiple measures, all other factors but the contingency should be held constant. This is, however, not easy to realize in our paradigm. Specifically, although stimuli were presented in a pseudorandomized fixed order, the number of received CS++, CS+, and CS− in world B (modification phase) differed between participants depending on their behaviour. Further, the fixed and for all participants same reinforcement plan induced the possibility that the changes in the contingency were less noticeable for some participants (i.e., those who selected few 10 m steps in world A) than for others (i.e., those who selected only 10 m steps in world A). The implementation of a yoked control group via a matched-pairs design (for a good example see Ref.^45^) seems promising to address this issue. With respect to our paradigm, this would mean that each participant of one group (contingent movement-pain association) would be paired with a participant of another group (no contingent movement-pain association) and that both receive the same relative amount of heat pain stimulation but with different contingencies. Such a design is, however, complicated to realize as the total number of applied stimuli depends on the participant’s actual behaviour (see above). This behaviour is unknown in the beginning and prevents us from adapting the ratio of the three stimuli in advance. To fix the numbers of movements via overt instructions might solve this issue, however, this would preclude the opportunity to study associative learning mechanisms^14^. Another option which could be worth to explore is the inclusion of an additional control group that receives no pain in the modification phase. Although we did not consider this as more ecologically valid than the current modification phase with unpredictable pain, it would allow for more insights into the relative strengths of effects of contingent vs. random vs. not any pain on pain-related approach behaviour and especially into the question under which circumstances the previously learned associations between certain movements and certain pain stimuli can be modified and ultimately extinguished.

Fifth, the use of Virtual Reality requires further consideration. For treatment purposes Virtual Reality has been successfully applied to different chronic pain conditions (e.g., headache^46^; fibromyalgia^47^; for review see Ref.^18^). However, observed reductions in pain intensity were ascribed primarily to one of three mechanisms: distraction, neuromodulation, or graded exposure^21^. Whether effects persist beyond the momentary use of the application remained unclear^48,49^. In contrast to this line of research that primarily focused on pain intensity, a recent meta-analysis suggests that Virtual Reality paradigms are also able to reduce fear of movements, and disability (e.g., Ref.^50^). In respect to pain-associated approach-avoidance behaviour, Virtual Reality would, for instance, allow researchers to give participants targeted feedback about their own movements. This raises the possibility to modify this feedback in a way that leads to gradual normalization of pathologically restricted movement ranges^24^. The transfer of our paradigm to groups of chronic pain patients may represent the next step to gain more insights into how associative and operant learning mechanisms differ between chronic pain patients and healthy controls, which could then be adapted for treatment purposes. Another important point is that 2D Virtual Reality applications as used in our study (projections on a 2D wallscreen) were suggested to induce lower immersion than 3D techniques (mostly implemented with head mounted displays, HDMs^51^). Thus, especially when it comes to the development of treatments, the use of HDMs might present a further improvement to this paradigm.

A sixth and last promising line of further development of the paradigm is the inclusion of additional trait measures to investigate how individual differences in trait constructs relate to individual differences in the respective associative and operant learning mechanisms. Candidate constructs include trait fear of pain, pain catastrophizing, kinesiophobia, trait anxiety, anxiety sensitivity, and depression all of which can be assessed using standardized questionnaires like the Fear of Pain Questionnaire (FPQ^52^), the Pain Catastrophizing questionnaire (PCS^53^), the Tampa Scale of Kinesiophobia (TSK^54^), the State Trait-Anxiety-Depression-Inventory (STADI^55^), the Anxiety Sensitivity Index (ASI^56^), and the Beck Depression Inventory (BDI-II^57^). Although the focus of this proof-of-concept study was the evaluation of our paradigm’s potential for further research, and we therefore limited our analyses to group-average effects, a more comprehensive investigation of individual differences in respective mechanisms might be valuable, for instance, to identify individual vulnerability factors^58^.

## Conclusion

Our study proposes an experimental Virtual Reality paradigm that allows researchers to investigate pain-associated approach behaviour and related psychological and physiological mechanisms in a controlled laboratory setting. We manipulated the contingency between three types of movements and three types of heat pain stimuli in a group of healthy participants and observed that changes in movement-pain contingencies resulted in changes of pain-associated approach behaviour, self-rated fear of movement-related pain and pain expectancy, as well as in alterations of physiological arousal (electrodermal activity). Although the observed effects require replication in a larger and more representative sample, our results provide insights into the fast adaptation of approach behaviour in the presence of acute pain. The here introduced paradigm can be adapted in various ways to gain new insights into the development and maintenance of chronic pain.

### Supplementary Information

Supplementary Information.

## Data Availability

As this is the first study of a larger research project (details are publicly available under https://osf.io/zh3ak/) and data acquisition is still ongoing, data will be shared via OSF after data collection is completed. Any analysis code, technical support, and the experimental paradigm can be accessed from the first author (kirsten.hilger@uni-wuerzburg.de) upon request.
