# Supplementary material for: Virtual reality to understand pain-associated approach behaviour: a proof-of-concept study
Source: Sci Rep. 2023 Aug 23;13:13799. doi: 10.1038/s41598-023-40789-z (PMC10447531; doi:10.1038/s41598-023-40789-z)
Supplement: Supplementary file 1 — Supplementary Information. [file 41598_2023_40789_MOESM1_ESM.pdf]

SUPPLEMENTARY INFORMATION

Virtual Reality to understand Pain-Associated Approach Behaviour: A Proof-of-Concept Study

Kirsten Hilger[a*], Anne-Sophie Häge[a], Christina Zedler[a], Michael Jost[a], Paul Pauli[a]

[a]*Department of Psychology I, University of Würzburg, Würzburg, Germany*

ORCID:

Kirsten Hilger: 0000-0003-3940-5884

Paul Pauli: 0000-0003-0692-6720

***Correspondence:**

Kirsten Hilger

Department of Psychology I

Marcusstr. 9-11

D-97070 Würzburg

Phone: +49 931 31-81141

Mail: kirsten.hilger@uni-wuerzburg.de

Supplementary Table S1. Descriptive statistics of exploratively assessed variables.

	M (SD)	*Min*	*Max*	*M (SD)*	*Min*	*Max*
	During acquisition phase (World A)			During modification phase (World B)		
Total Number of 5m Steps	31.6 (21.4)	0	76	10.7 (9.8)	0	36
Total Number of 1m Steps	8.0 (4.0)	0	37	4.0 (5.7)	0	27
SCR to 5m Steps	0.7 (0.5)	0.1	3.0	0.8 (0.8)	0.1	4.2
SCR to 1m Steps	0.4 (0.3)	0	1.1	0.6 (0.8)	0.1	3.1

Skin conductance responses (SCR) and skin conductance levels (SCL) are depicted in mS (micro Siemens). All above listed measures were averaged across trials. Trial-specific primary outcome measures are depicted in Supplementary Figures 1 and 2.

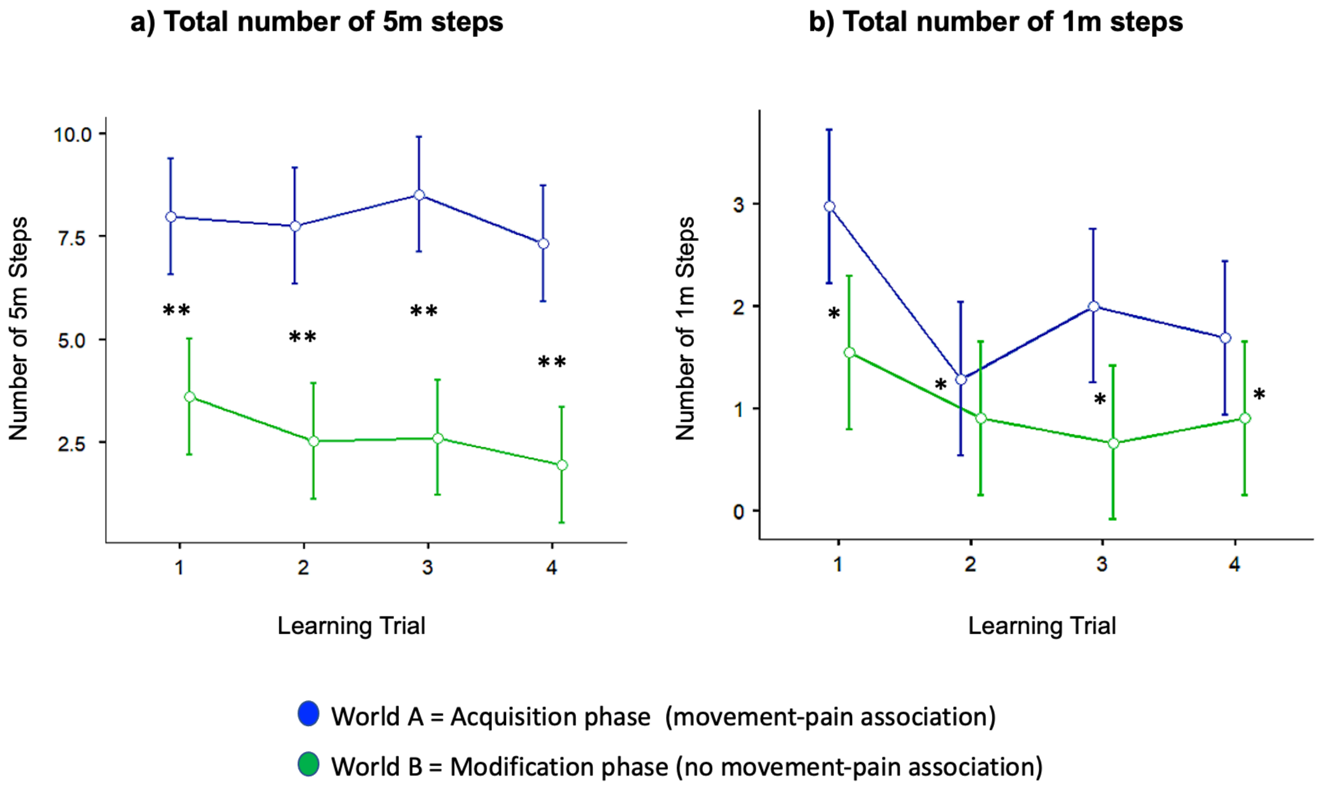

Supplementary Figure S1. Total number of 5m (a) and 1m (b) steps over the four learning trials in the acquisition phase (world A, contingent movement-pain association) and in the modification phases (world B, no movement-pain association). The graphs illustrate the two-factorial repeated-measures ANOVAs conducted as additional exploratory post-hoc analyses with factors learning trial and phase (acquisition vs. modification). For 5m steps (CS+) a significant main effect was observed for the factor world ($F(1,41) = 49.66$, $p < .001$, $\eta^2p = .548$) and the factor trial ($F(3,123) = 4.59$, $p = .004$, $\eta^2p = .101$), while there was no interaction between both factors ($F(2.17,88.78) = 1.66$, $p = .195$, $\eta^2p = .039$). Similarly, for 1m steps (CS-) significant main effects were observed for factor world ($F(1,41) = 11.01$, $p = .002$, $\eta^2p = .212$) and trial ($F(2.49,102.22) = 8.71$, $p < .001$, $\eta^2p = .175$), while the interaction between both factors reached no significance ($F(2.19,89.85) = 1.57$, $p = .213$, $\eta^2p = .037$). Error bars indicate 95% confidence intervals. *$p < .05$ and **$p < .01$ indicating statistical significance of post-hoc paired *t*-tests (uncorrected for multiple comparisons), i.e., directly comparing two learning trials between both phases (acquisition vs. modification phase).

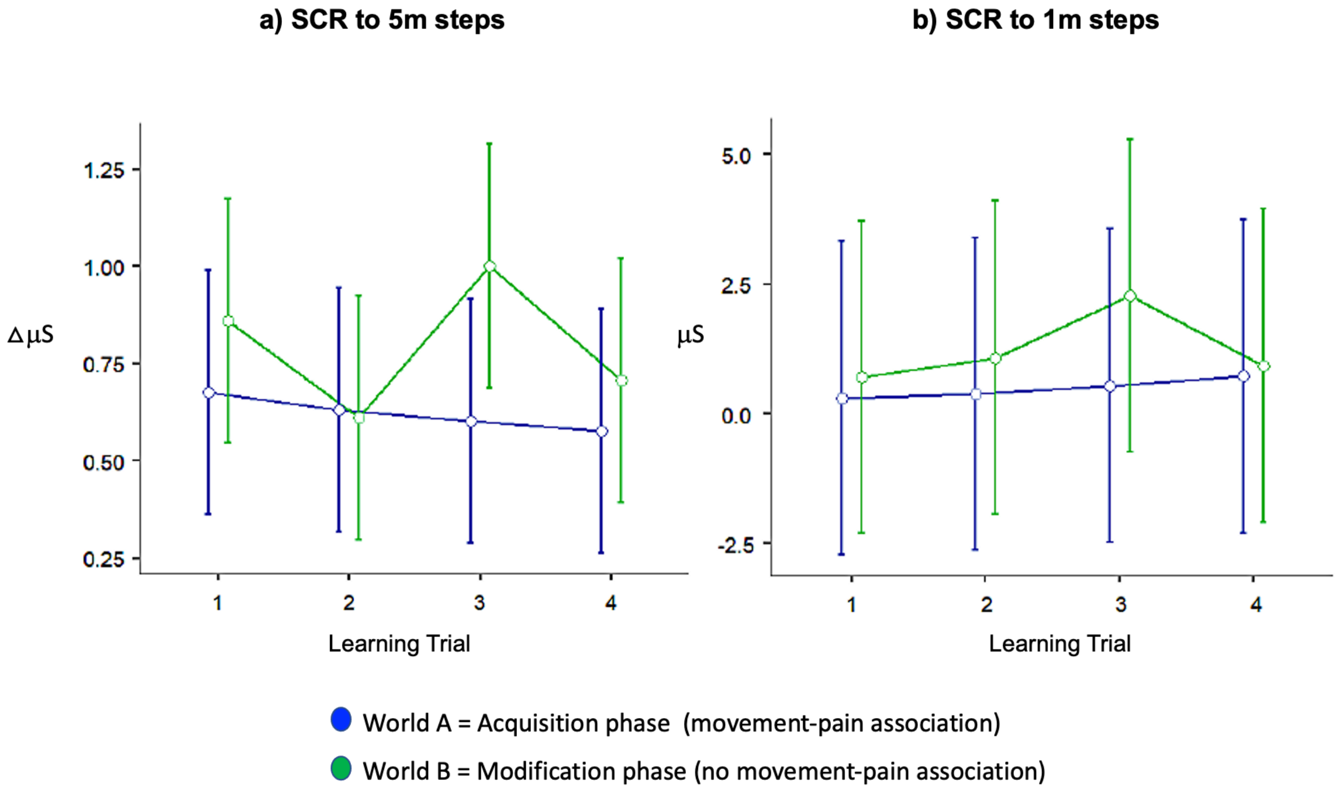

Supplementary Figure S2. Skin conductance responses to 5m (a) and 1m (b) steps over the four learning trials in the acquisition phase (world A, contingent movement-pain association) and in the modification phases (world B, no movement-pain association). The graphs illustrate the two-factorial repeated-measures ANOVAs conducted as additional exploratory post-hoc analyses with factors learning trial and world (acquisition vs. modification phase). Skin conductance responses (SCR) are depicted in mS (micro Siemens). No significant interaction or main effects were observed (all $p > .05$). Error bars indicate 95% confidence intervals.

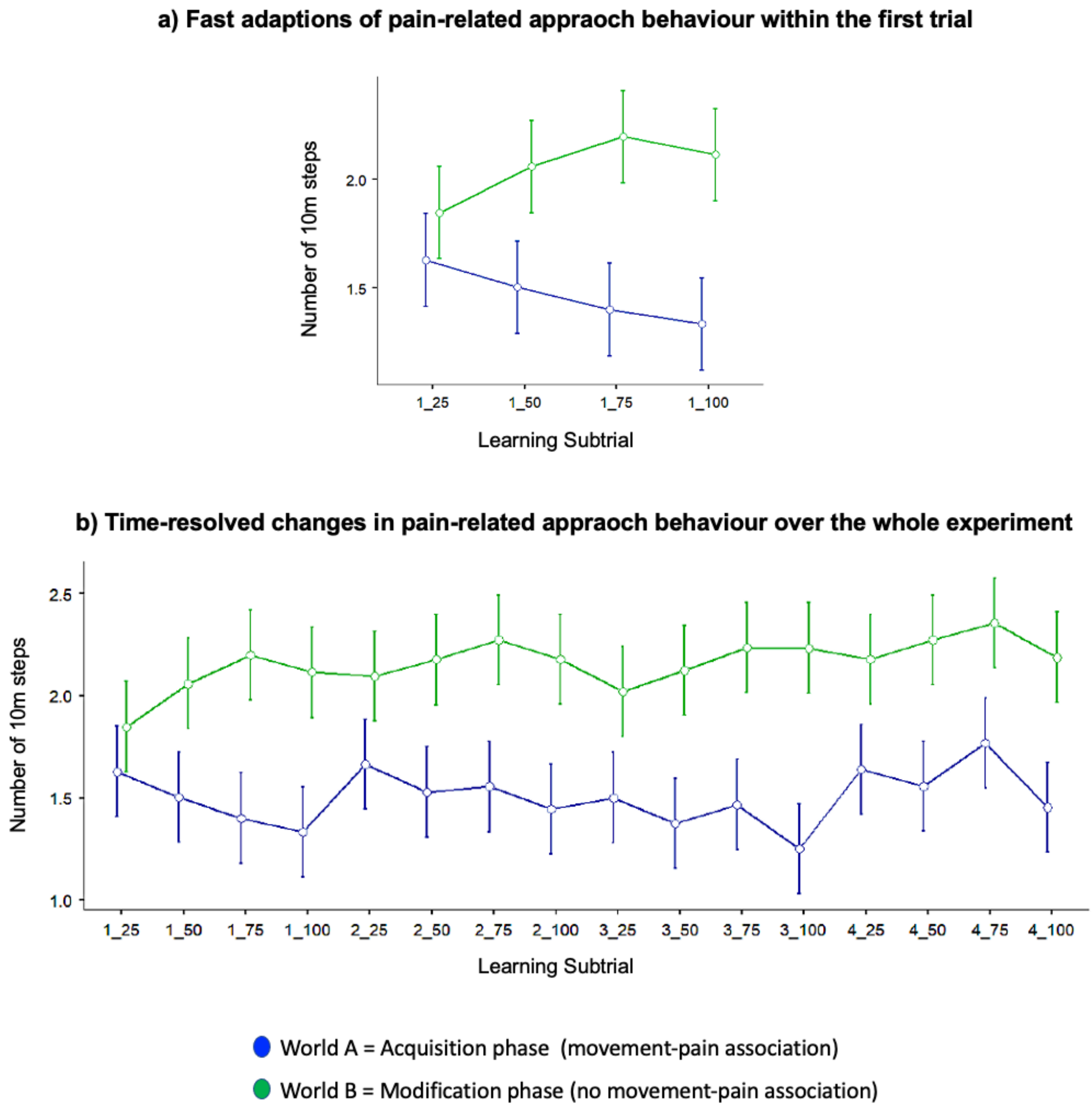

Supplementary Figure S3. Time-resolved changes in pain-related approach behavior within the first learning trial (a) and over the course of the whole experiment (b). For this post-hoc analyses each 100m path in VR was subdivided into four 25m paths (subtrials). The graphs illustrate the two-factorial repeated-measures ANOVAs conducted as additional exploratory post-hoc analyses with factors learning subtrial and world (acquisition vs. modification phase). Error bars indicate 95% confidence interval. See Results section 3.3.1 for further details.

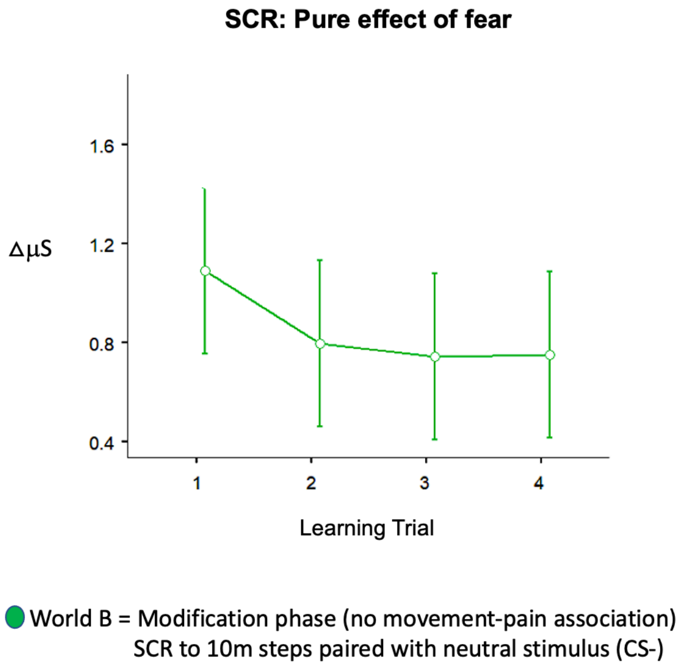

Supplementary Figure S4. Skin conductance responses to 10m steps associated with neutral heat stimuli (CS-) in world B. To illustrate the pure effect of fear in the SCR, i.e., free from any physiological reaction to heat pain, only those SCRs to 10m steps were extracted and analyzed that were followed by a neutral heat stimulus (CS-) in world B. Error bars indicate 95% confidence intervals.

Supplementary Results

Unhappiness rated via the Self-Assessment Manikin Scale (SAMS) was higher after the modification phase than after the acquisition phase ($t(41) = 5.088$, $p < .001$, Cohens $d = .785$), while no significant differences were observed for arousal and perceived control (arousal: $W(41) = 593.0$, $p = .078$, $r = .313$; control: $W(41) = 591.0$, $p = .082$, $r = .309$). However, in both cases we observed non-significant trends towards lower arousal and lower perceived control after the modification phase (Table 1).
